# The Catalytic Mechanisms of the Reactions between Tryptophan Indole-Lyase and Nonstandard Substrates: The Role of the Ionic State of the Catalytic Group Accepting the Cα Proton of the Substrate

**DOI:** 10.32607/20758251-2019-11-3-82-88

**Published:** 2019

**Authors:** N. G. Faleev, M. A. Tsvetikova, O. I. Gogoleva, V. V. Kulikova, S. V. Revtovich, K. A. Kochetkov

**Affiliations:** Nesmeyanov Institute of Organoelement Compounds, Russian Academy of Sciences, Vavilova Str. 28 , Moscow, 119991, Russia; Engelhardt Institute of Molecular Biology, Russian Academy of Sciences, Vavilova Str. 32, Moscow, 119991, Russia; Mendeleev University of Chemical Technology, Miusskaya Sq. 9, Moscow, 125047, Russia

**Keywords:** tryptophan indole-lyase, mechanism, kinetics, L-serine, β-chloro-L-alanine

## Abstract

In the reaction between tryptophan indole-lyase (TIL) and a substrate containing a bad leaving group (L-serine), general acid catalysis is required for the group's elimination. During this stage, the proton originally bound to the C_α_ atom of the substrate is transferred to the leaving group, which is eliminated as a water molecule. As a result, the basic group that had accepted the C_α_ proton at the previous stage has to be involved in the catalytic stage following the elimination in its basic form. On the other hand, when the substrate contains a good leaving group (β-chloro-L-alanine), general acid catalysis is not needed at the elimination stage and cannot be implemented, because there are no functional groups in enzymes whose acidity is strong enough to protonate the elimination of a base as weak as Cl- anion. Consequently, the group that had accepted the C_α_ proton does not lose its additional proton during the elimination stage and should take part in the subsequent stage in its acidic (not basic) form. To shed light on the mechanistic consequences of the changes in the ionic state of this group, we have considered the pH dependencies of the main kinetic parameters for the reactions of TIL with L-serine and β-chloro-L-alanine and the kinetic isotope effects brought about by replacement of the ordinary water used as a solvent with 2H_2_O. We have found that in the reaction between TIL and β-chloro-L-alanine, the aminoacrylate hydrolysis stage is sensitive to the solvent isotope effect, while in the reaction with L-serine it is not. We have concluded that in the first reaction, the functional group containing an additional proton fulfills a definite catalytic function, whereas in the reaction with L-serine, when the additional proton is absent, the mechanism of hydrolysis of the aminoacrylate intermediate should be fundamentally different. Possible mechanisms were considered.

## INTRODUCTION

In studies focused on enzymic mechanisms, the basic notion frequently taken into account is that completion of any stage of the process creates favorable chemical and conformational prerequisites for the subsequent stages [1]. In this context, the mechanisms of enzymes displaying broad substrate specificities are of considerable interest, since some situations arising in the active site depending on the chemical nature of the substrate may violate the aforementioned principle. Tryptophan indole-lyase (TIL), also known as tryptophanase (EC 4.1.99.1), is a pyridoxal-5’-phosphate (PLP)-dependent enzyme catalyzing the reversible α,β-elimination of L-tryptophan with the formation of indole and ammonium pyruvate.

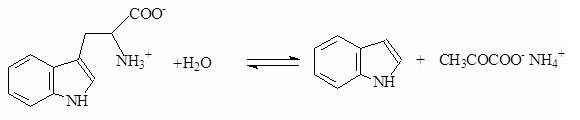



The other substrates of TIL are tryptophan analogs substituted at various positions of the indole ring [2, 3], benzimidazole analogs of tryptophan [4], as well as amino acids containing suitable leaving groups at the β-carbon atom, including S-(o-nitrophenyl)-L-cysteine (SOPC) [5], S-alkyl-L-cysteine analogs [6], β-chloro-Lalanine [5], and L-serine [6] and O-acyl-L-serines [7].

The three-dimensional structure was established by X-ray analysis for TIL from Escherichia coli [8, 9, 10] and for the enzyme from Proteus vulgaris [11]. The catalytic mechanism of TIL was studied in detail in [12-16]; the role of specific residues in the mechanism of TIL was elucidated in [17-20].

Scheme 1 shows the catalytic mechanism of TIL with its natural substrate, L-tryptophan, which is in agreement with the known X-ray and kinetic data. The key stages in this mechanism involve the abstraction of the α-proton of external aldimine under the action of the side amino group of the lysine 270 residue, and subsequent elimination of the side indole group assisted by proton transfer from phenol hydroxyl of the tyrosine 74 residue to the 3-position of the leaving indole group. According to the data reported in [16], proton transfer and breaking of the C–C bond proceed almost simultaneously. It was determined in [21] that enzymic decomposition of L-tryptophan is accompanied by a considerable intramolecular transfer of the Cα proton of the substrate to the 3-position of the indole that has been formed. Since the lysine 270 and tyrosine 74 residues are far apart from each other and are located on opposite sides of the cofactor plane, direct transfer of a proton between them seems improbable. Therefore, the observed intramolecular transfer [21] might be a result of the existence of a chain of hydrogen bonds between several residues, which renders the observed transfer possible. Convincing X-ray evidence of the existence of such a chain was presented in [20]. In α,β-elimination reactions with substrates containing bad leaving groups (e.g., L-serine), general acid catalysis is required at the stage of the leavinggroup elimination. During this stage, formal transfer of a proton (either directly or through the chain of hydrogen bonds) from the Cα position of the substrate to the leaving group takes place; the latter is eliminated in the form of the respective conjugated acid. As a result, the base that has originally accepted the α-proton should appear as the respective conjugated base once the leaving group has been eliminated. β-Chloro-L-alanine is known to be a good substrate for α,β- and α,γ-eliminating lyases. In the reactions with this substrate, the role of the leaving group is played by a chlorine anion. No general acid catalysis is needed with such a leaving group; in this case, it cannot even be implemented, since the enzymes carry no functional groups whose acidities are strong enough for the acids to give away their protons to a base as weak as the chlorine anion. Consequently, the catalytic group that had originally accepted the α-proton from the substrate should appear in its acidic, rather than basic, form at the following stage. We believe that it is of considerable interest what mechanistic consequences the change in the ionic state of this group has. Two possibilities seem plausible: (1) the emergence of a new acidic group in the pH profile of kinetic parameters, which is associated with the necessity of a transition of the group that has accepted the α-proton into its basic form; (2) changes in the mechanism of the stage(s) following the elimination brought about by the changes in the ionic state of the aforementioned catalytic group. In the present work, we attempted to shed light on this question by considering the pH dependencies of the main kinetic parameters of the reactions of TIL from Escherichia coli with L-serine and β-chloro-L-alanine, as well as the kinetic isotope effects resulting from the replacement of ordinary water, as a solvent, for 2H_2_O.

**Scheme:**
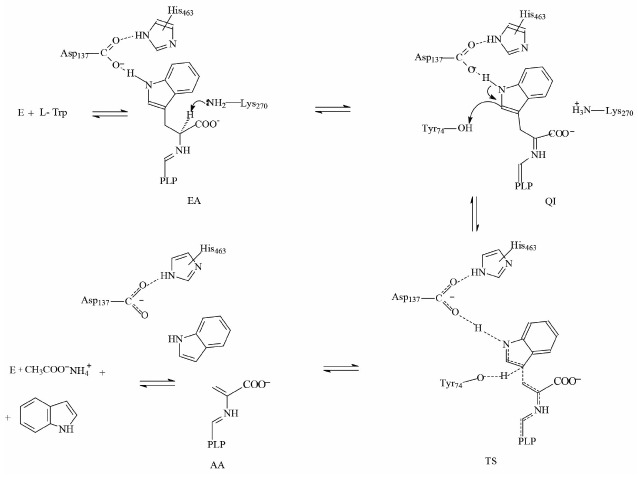
The principal mechanism of the reaction between TIL and L-tryptophan, which is consistent with [12-18]. E – internal aldimine; EA – external aldimine; QI – quinonoid intermediate; TS – transition state; and AA – aminoacrylate

## EXPERIMENTAL

The reagents used in this work were purchased from Sigma-Aldrich. The isotopic purity of 2H_2_O was 99%.

**Enzyme**

Tryptophan indole-lyase was isolated from E. coli JM101 cells containing plasmid pMD6 with the E. coli tnaA gene, as described in [22]. Enzyme concentrations were estimated from the absorbance of the holoenzyme at 278 nm (A_1%_ = 9.19) [23] using a subunit molar mass of 52 kDa [24].

The activity of TIL was determined using S-o-nitrophenyl- L-cysteine (SOPC) as a substrate. The reaction mixture contained 0.6 mmol SOPC, the enzyme, 0.12 M potassium phosphate buffer (pH 7.8), 3 mM dithiothreitol, 0.06 mM PLP, and 10% glycerol. Activity was measured at 30°C according to the decline in SOPC absorbance at 370 nm (ε = -1860 M^-1^min^-1^). One unit of activity was assumed equal to the amount of enzyme catalyzing the decomposition of one micromole of SOPC per minute under standard conditions. SOPC was synthesized as described in [25].

Steady-state kinetic measurements were performed at 30°C using the lactatedehydrogenase (LDH) coupled assay. Reaction mixtures contained 0.2 mM NADH, 8 units of LDH, and 0.2 μM TIL in 0.1 M potassium phosphate or borate buffer solutions in the presence of 0.1mM PLP at various pH and substrate concentrations. The reaction rates were determined at 30°C according to the decline in absorbance of NADH at 340 nm (ε = - 6220 M^-1^cm^-1^).

**Determination of the solvent kinetic isotope effect (SKIE)**

The potassium phosphate buffer solution (20 ml, pH 8.2) was evaporated to dryness in vacuum. The residue was vacuum-dried over CaCl_2_ and dissolved in 20 ml of 2H_2_O. The obtained buffer solution was used for kinetic studies under conditions analogous to those described earlier for solutions in water.

Comparing the kinetic parameters for the reactions in water and 2H_2_O allowed us to collect the data presented in Table. The steady-state kinetic data were analyzed using the Cleland’s FORTRAN programs [26].

## RESULTS AND DISCUSSION

In the present work, we have studied the pH dependencies of the main kinetic parameters for the reactions of TIL with L-serine and β-chloro-L-alanine. The results were compared with the literature data for the reaction of TIL with its natural substrate, L-tryptophan [13]. For this reaction, the pH dependence of V/K can be described by equation (1) with two pKs equal to 7.6 and 6.0.

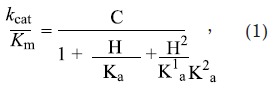



where pK_a_ = 7.6 ± 0.09, K_b_ = 6.0 ± 0.2.

The value of 7.6 can be ascribed to the amino group of the Lys270 residue, which is responsible for the abstraction of the C_α_ proton of the external aldimine, whereas the pK equal to 6.0 can be ascribed to the side group of Asp137 interacting with nitrogen of the indole moiety at the stage of substrate binding [15, 17], which leads to activation of the indole group as a leaving group.

**Fig. 1:**
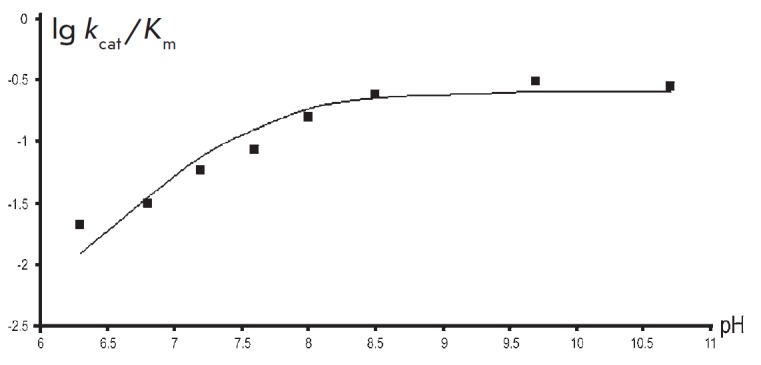
The pH dependence of V/K for the reaction between TIL and L-serine. The points denote the experimentally determined values obtained by data-fitting to the Michaelis–Menten equation, while the curve was plotted by fitting the resulting values using Eq. (2) where pK_a_ = 7.6

We have shown that pH dependence of V/K for the reaction with L-serine (Fig. 1) could be described by equation (2) with one pK equal to 7.6.

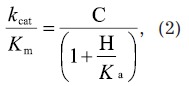



where pK_a_ = 7.6 ± 0.1.

A conclusion can be drawn that ionization of the acidic group of Asp137, which takes part in the activation of the leaving indole group in the reaction with the natural substrate, is not reflected in the pH dependence for the reaction with L-serine. We may assume that serine conformation in the active site is analogous to that of tryptophan in the sense that the position of hydroxylic oxygen strictly corresponds to the position of the Cγ atom of the indole ring. In this case, according to the X-ray data [20], hydroxylic oxygen of L-serine should be located in close proximity to the phenol group of the Tyr74 residue, which is connected to the amino group of the Lys270 residue by a chain of hydrogen bonds [20]. In the course of α,β-elimination, a proton from the ammonium group of Lys270 is transferred to Tyr74 through the chain of hydrogen bonds. The Tyr74 residue donates its own proton to the hydroxylic group of serine, which is eliminated as water. The ionic states of all the participants in this process, except for Lys270, remain unchanged, and the whole process may be considered a formal transfer of a proton from Lys270 to the leaving group. It seems probable that, in the pH range under study, the phenol group of the Tyr74 residue remains in its acidic form, which is needed for the reaction to proceed. This explains the absence of the respective pK in the pH dependence.

**Fig. 2:**
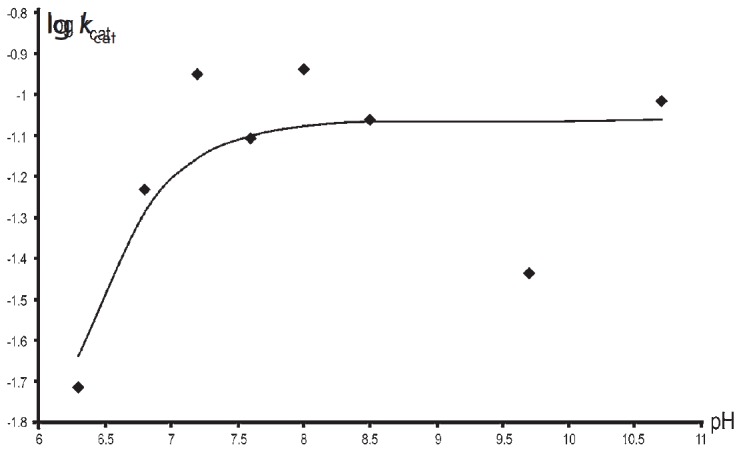
The pH dependence of V for the reaction between TIL and L-serine. The points are the experimentally determined values obtained by fitting the data to the Michaelis–Menten equation, while the curve was plotted by fitting the resulting values using Eq. (1), where pK_a_ = pK_b_ = 6.3

**Fig. 3:**
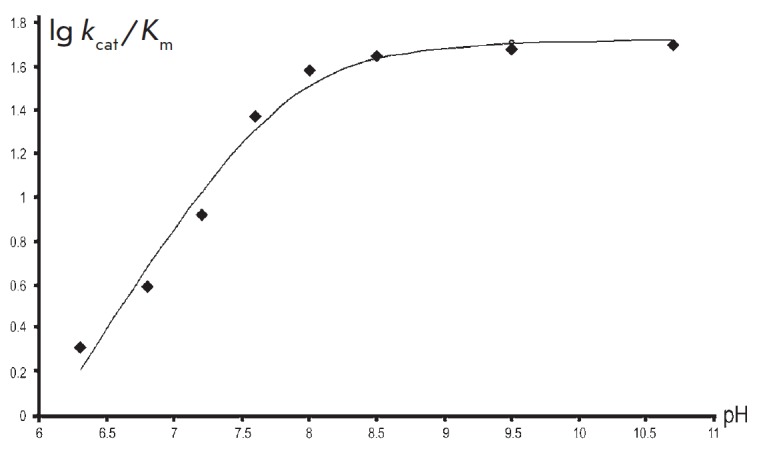
The pH dependence of V/K for the reaction between TIL and β-chloro-L-alanine. The points are the experimentally determined values obtained by data-fitting to the Michaeli–Menten equation, while the curve was plotted by fitting the resulting values using Eq. (2), where pKa = 7.8

**Fig. 4:**
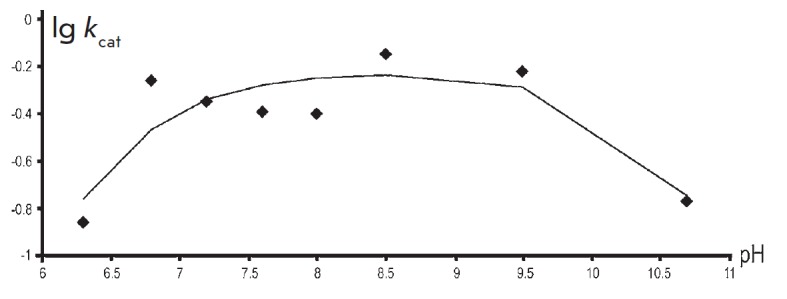
The pH dependence of V for the reaction between TIL and β-chloro-L-alanine. The points are the experimentally determined values obtained by data-fitting to the Michaelis–Menten equation, while the curve was plotted by fitting the resulting values using Eq. (3), where pK_a_ = 6.7, pK_b_ = 10.3

Figure 2 shows the pH dependence of k_cat_ for the reaction of TIL with L-serine, which can be described by an equation with two similar pKs (Eq. (1), where pK_a_ = pK_b_ = 6.3 ± 0.1). At the same time, it was established for the reaction of TIL with L-tryptophan [13] that kcat is independent of pH, thus providing evidence for a protonation mechanism in which the substrate binds only to the correctly protonated enzyme form. As a result, the enzyme–substrate complex forms, being inaccessible to protons from the environment. It seems probable that in the reaction with L-serine containing a small side group, the latter occupies less space in the active site. Therefore, hydroxonium cations from the external solvent are able to penetrate into the enzyme–substrate complex and protonate certain functional groups, thus making the reaction impossible. We have shown that for the reaction of TIL with β-chloro-L-alanine the pH dependence of V/K (Fig. 3) is virtually identical to a similar dependence for the reaction with L-serine. It can be described by an equalgtion with one pK_a_ (Eq. (2)) equal to 7.8 ± 0.1. Meanwhile, the pH dependence of V has a fundamentally different, bell-shaped appearance (Fig. 4) and can be described by Eq. (3):

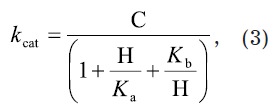



where pK_a_ = 6.7 ± 0.2; pK_b_ = 10.3 ± 0.2.

As it was mentioned above, the reaction with β-chloro-L-alanine is most likely to proceed without activation of the leaving group, which is eliminated as a chlorine anion. As a consequence, the situation in the active site immediately after the elimination of Clshould fundamentally differ from that in the reaction with L-serine, because the proton originally bound to the α-carbon atom of the substrate in the reaction of β-chloro-L-alanine remains in the active site, while it is withdrawn from the active site together with the leaving group in the reaction with L-serine. We may assume that the pKb value = 10.3, which was observed in the pH profile of V for the reaction of β-chloro-Lalanine, reflects the acidic dissociation of exactly this additional proton in the enzyme–substrate complex. The observed decrease in V can be associated with a given catalytic function fulfilled by the respective acidic group during chemical transformations following the elimination of the chlorine anion.

**Table 1:** The solvent isotope effect on the kinetic parameters of the TIL reactions with L-serine and β-chloro-L-alanine

Substrate	Parameter	SIE
L-serine	V/K	3.5 ± 0.5
L-serine	V	0.8 ± 0.2
β-chloro-L-alanine	V/K	2.2 ± 0.5
β-chloro-L-alanine	V	3.6 ± 1.2

In order to conduct a detailed study of the roles played by various elementary stages in the mechanisms of reactions with nonstandard substrates, we examined the kinetics of the reactions of TIL with β-chloro-L-alanine and L-serine in water and ^2^H_2_O in the optimal pH range and determined the solvent isotope effects on the main kinetic parameters. These results are presented in Table. Unlike in the reaction with a natural substrate, reactions of TIL with L-serine and with β-chloro-L-alanine proceed only in the direction of substrate decomposition, but not their synthesis. Thus, the α,β-elimination yielding an aminoacrylate intermediate in the active site is irreversible in this reaction. Taking this fact into account, we considered the mechanisms of both reactions under the following kinetic scheme (scheme 2):

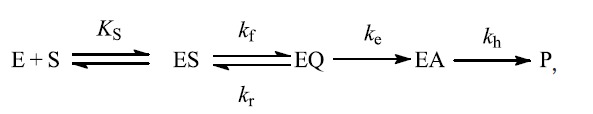



where E is the internal aldimine, ES is the external aldimine, EQ is the quinonoid intermediate, EA is the aminoacrylate complex, and P is the reaction product (pyruvate).

For the presented kinetic scheme, the main kinetic parameters are described by Eqs. (4) and (5).

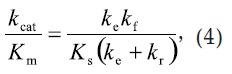

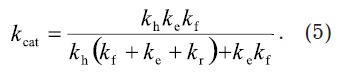





One can see that the solvent isotope effect on V/K for the reaction between TIL and L-serine is equal to 3.5 (see Table). Among the constants in Eq. (4), k_e_ is not isotope-sensitive if the abstraction of the C_α_ proton under the action of the Lys270 amino group occurs directly. On the contrary, the k_r_ value should be isotopesensitive because this constant refers to the return of a proton to the C_α_ atom of the quinonoid intermediate under the action of the ammonium group of Lys270, which contains at least two deuterons in ^2^H_2_O, and even three deuterons if isotopic exchange with the solvent proceeds sufficiently fast. However, as it follows from Eq. (4), this effect should accelerate the reaction in ^2^H_2_O, whereas in fact we observed that it slowed down. Hence, a conclusion can be drawn that elimination of the leaving hydroxylic group is the only stage determining the observed solvent isotope effect, since it assumes that a proton is transferred from Lys270 to Tyr74 through the chain of hydrogen bonds, and then to the hydroxylic oxygen. When ordinary water used as a solvent is replaced with ^2^H_2_O, all the protons involved in this transfer are exchanged for deuterons and the process is expected to slow down. It follows from the data presented in Table that the solvent isotope effect on V within the experimental error does not differ from unity. This probably results from the fact that a new constant, k_h_, appears in Eq. (5) describing k_cat_; Eq. (4) did not contain this constant. It determines the rate of aminoacrylate hydrolysis. It is evident that when k_h_ (k_f_ + k_t_ +k_r_) < < kekf, the kcat value should be equal to the k_h_ (k_cat_ ~ k_h_) value. The kh constant is apparently rate-limiting; on the other hand, it is insensitive to the solvent isotope effect. In the case of the TIL reaction with β-chloro-L-alanine, the elimination of the leaving group should not be accompanied by proton transfer to the chlorine anion being eliminated. Consequently, the stage described by the ke constant should not be isotope- sensitive. Everything that has been said about the k_f_ and kr constants in the reaction with L-serine should also be true for the reaction with β-chloro-L-alanine. Therefore, it seems reasonable to suggest that there is no solvent isotope effect on the V/K parameter. However, an isotope effect equal to 2.2 is in fact observed. A plausible explanation is that the stage of C_α_-proton abstraction (k_f_) may proceed not directly but through a water molecule (or molecules), which is expected to reduce k_f_ when the solvent is changed from water to ^2^H_2_O. A similar phenomenon can also take place in the reaction with L-serine. In this case, the solvent isotope effect on V/K, observed for this reaction, can be associated not only with the stage of aminoacrylate formation.

**Fig. 5:**
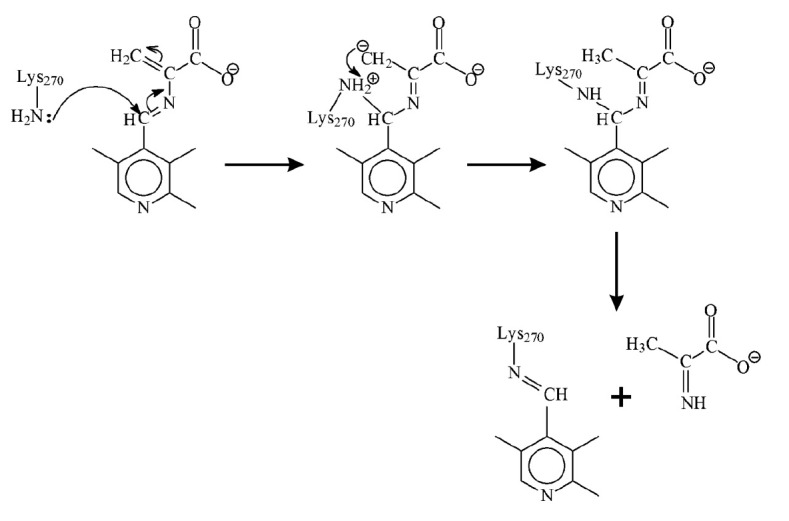
The possible scheme of aminoacrylate hydrolysis in the reaction between TIL and L-serine

**Fig. 6:**
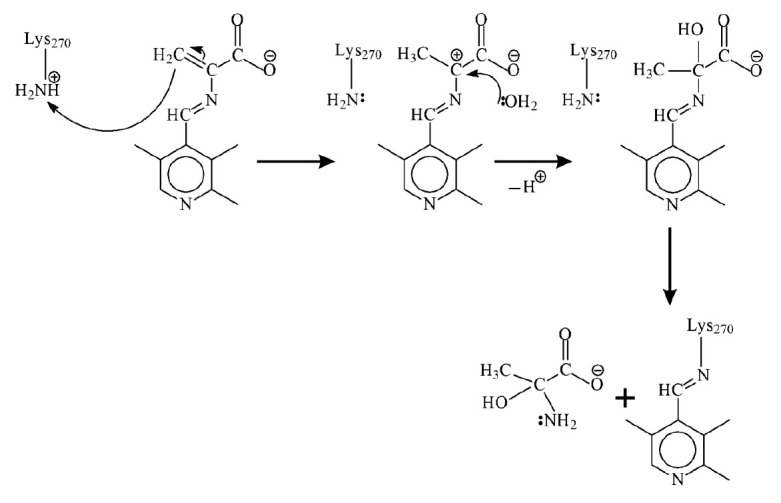
The possible scheme of aminoacrylate hydrolysis in the reaction between TIL and β-chloro-L-alanine

For the reaction with β-chloro-L-alanine, the solvent isotope effect on parameter V is equal to 3.6 (see Table). Therefore, the emergence of the rate of aminoacrylate hydrolysis (kh) in Eq. (5) considerably increases the isotope effect, contrary to its decline in the L-serine reaction. It is fair to conclude that aminoacrylate hydrolysis is an isotope-sensitive stage in the reaction with β-chloro-L-alanine; the hydrolysis mechanism differs significantly from that in the reaction with L-serine. In the reaction with L-serine, the amino group of Lys270 exists in its basic form at the stage of aminoacrylate hydrolysis. The attack of the lysine amino group at the aldimine double bond of the aminoacrylate intermediate is probably the rate-limiting stage of hydrolysis (see Fig. 5). Since no transfer of protons that could be exchanged for deuterons accompanies the limiting stage, the hydrolysis should be insensitive to solvent replacement. On the other hand, in the reaction with β-chloro-L-alanine, a similar limiting stage cannot be implemented because the side amino group of Lys270 is present in its acidic---ammonium---form containing the additional proton. The ammonium group can donate this additional proton to the methylene group of aminoacrylate, most probably through the chain of hydrogen bonds (see Fig. 6). Since the protons of the ammonium group and those participating in the chain of hydrogen bonds can undergo isotopic exchange with the solvent, aminoacrylate hydrolysis should be an isotope-sensitive stage, which was actually observed.

## CONCLUSIONS

Hence, the results of our work show that the changes in the nucleophilic nature of the leaving group in TIL substrates may alter not only the mechanism of elimination of the leaving group, but also the mechanism of the subsequent stage of aminoacrylate hydrolysis.

## Abbreviations

TIL: tryptophan indole-lyase
PLP: pyridoxal-5’-phosphate
SOPC: S-o-nitrophenyl-L-cysteine
LDH: lactate dehydrogenase
NADH: nicotinamide-adenine dinucleotide
SKIE: solvent kinetic isotope effect
